# Extremely discrepant mutation spectrum of *SLC26A4 *between Chinese patients with isolated Mondini deformity and enlarged vestibular aqueduct

**DOI:** 10.1186/1479-5876-9-167

**Published:** 2011-09-30

**Authors:** Shasha Huang, Dongyi Han, Yongyi Yuan, Guojian Wang, Dongyang Kang, Xin Zhang, Xiaofei Yan, Xiaoxiao Meng, Min Dong, Pu Dai

**Affiliations:** 1 Department of Otolaryngology, PLA General Hospital, Beijing, People's Republic of China; 2 Medical college, Nankai University, Tianjin, People's Republic of China

**Keywords:** *SLC26A4*, hearing loss, Mondini dysplasia, enlarged vestibular aqueduct, inner ear malformation.

## Abstract

**Background:**

Mutations in *SLC26A4 *cause Pendred syndrome (hearing loss with goiter) or DFNB4 (non-syndromic hearing loss with inner ear malformation, such as enlarged vestibular aqueduct or Mondini deformity). The relationship between mutations in *SLC26A4 *and Mondini deformity without enlarged vestibular aqueduct has not been studied in any Chinese deaf population. The purpose of this study was to assess whether mutations in the *SLC26A4 *gene cause Mondini deformity without an enlarged vestibular aqueduct (isolated Mondini deformity) in a Chinese population.

**Methods:**

In total, 144 patients with sensorineural hearing loss were included and subjected to high-resolution temporal bone CT. Among them, 28 patients with isolated Mondini dysplasia (MD group), 50 patients with enlarged vestibular aqueduct with Mondini dysplasia (EVA with MD group), 50 patients with enlarged vestibular aqueduct without Mondini dysplasia (EVA group), and 16 patients with other types of inner ear malformations (IEM group) were identified. The coding exons of *SLC26A4 *were analyzed in all subjects.

**Results:**

DNA sequence analysis of *SLC26A4 *was performed in all 144 patients. In the different groups, the detection rate of the *SLC26A4 *mutation differed. In the isolated MD group, only one single allelic mutation in *SLC26A4 *was found in one patient (1/28, 3.6%). In the EVA with MD group, biallelic and monoallelic *SLC26A4 *mutations were identified in 46 patients (46/50, 92.0%) and three patients (3/50, 6.0%), respectively. Also, in the EVA group, biallelic and monoallelic *SLC26A4 *mutations were identified in 46 patients (46/50, 92.0%) and three patients (3/50, 6.0%), respectively. These percentages were identical to those in the EVA plus MD group. Only two patients carried monoallelic mutations of the *SLC26A4 *gene in the IEM group (2/16, 12.5%). There were significant differences in the frequency of *SLC26A4 *mutation among the groups (P < 0.001). The detection rate of *SLC26A4 *mutation in the isolated MD group was significantly lower than in the EVA group (with or without MD; P < 0.001), and there was no significant difference in the detection rate of *SLC26A4 *between the MD group and IEM group (P > 0.5).

**Conclusion:**

Although mutations in the *SLC26A4 *gene were frequently found in Chinese EVA patients with and without MD, there was no evidence to show a relationship between isolated MD and the *SLC26A4 *gene in the Chinese population examined. Hearing impairment in patients with isolated MD may be caused by factors other than mutations in the *SLC26A4 *gene.

## Introduction

Hearing impairment is the most common neurosensory disorder in humans with congenital or prelingual deafness, affecting about 1 in 300 to 1 in 1000 children [1-3]. Approximately half of cases have a genetic etiology, including syndromic and non-syndromic forms, with extraordinary genetic heterogeneity. Non-syndromic deafness accounts for 60-70% of inherited hearing impairment. It involves more than 100 different genes with autosomal dominant (DFNA), autosomal recessive (DFNB), X-linked (DFN), and maternal inheritance [4]; autosomal recessive is the most common. Mutations in the *SLC26A4 *gene are probably the second most common cause of inherited hearing loss, after *GJB2 *mutations, and are responsible for Pendred syndrome as well as DFNB4 (non-syndromic hearing loss with inner ear abnormalities, such as enlarged vestibular aqueduct (EVA) with or without Mondini dysplasia (MD)) [5,6].

Enlarged vestibular aqueduct syndrome (EVAS) and MD are the most frequently encountered radiographic anomalies of the inner ear. The most accepted criterion for definition of a large vestibular aqueduct is that suggested by Valvassori [7]: a vestibular aqueduct is considered to be enlarged if its diameter is > 1.5 mm at the midpoint between the common crus and the external aperture of the vestibular aqueduct on CT images. The imaging diagnostic criterion is differet about Mondini malformarion. Mondini first described by Carlo Mondini in 1791[8]detailed a small cochlea completing only one and a half turns and lacking a complete interscalar septum, addtionally, he described a bulbous endolymphatic sac and a dilated vestibular aqueduct. Since then, the term "Mondini" has been used as an umbrella term in clinical practice to describe almost any type of congenital cochlear deformity, included temporal bones with a common cavity and an incomplete interscalar septum of the cochlea. And a Mondini cochlea (isolated Mondini) occurs only when the two and a half turns of the cochlea are replaced by a single cavity in the apical region because of the absence of the interscalar septum [9-11]. It is usually bilateral and frequently associated with other inner ear malformations (IEMs) such as cystic expansion of the vestibules and semicircular canal dysplasia. Temporal bone abnormalities, ranging from isolated EVA to MD, have been causally linked to mutations in the anion transporter gene *SLC26A4*, which encodes the protein pendrin [5]. Pourova [12] found that 40.9% of Czech EVA/MD patients carried *SLC26A4 *mutations (27.3% had biallelic mutations and 13.6% had monoallelic mutations), and the detection rate of *SLC26A4 *mutations in Italian patients was 30% in Bogazzi's research [13]. These observations suggest that *SLC26A4 *mutations are frequently present in patients with EVA or MD. In the Chinese deaf population, *SLC26A4 *was also found to have a close relationship with the pathogenesis of DFNB4 [14,15], but no systematic study was applied to examine the role of *SLC26A4 *in the pathogenesis of isolated Mondini.

To understand the molecular etiology of isolated MD (MD without EVA) and provide possible genetic testing and counseling for hearing loss patients with isolated MD in China, we performed *SLC26A4 *sequence analysis in 28 patients with isolated MD, 50 patients with EVA and MD, 50 patients with isolated EVA, and 16 patients with other forms of IEM. In this study, the imaging diagnostic criterion of isolated Mondini is a cochlea of one and a half turns instead of the normal two and a half turns, comprising a normal basal turn and a cystic apex.

## Materials and methods

### Patients

**Figure 1:**
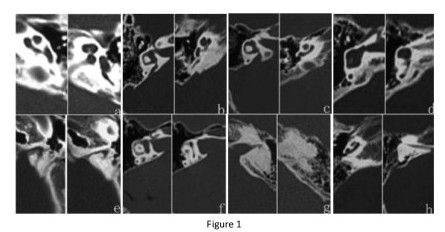
**Typical CT scan images of MD, EVA with MD, EVA, and other inner ear malformations**. a. MD; b. EVA with MD; c. EVA; d. common cavity (a cystic cavity representing the cochlea and vestibule without showing any differentiation into cochlea and vestibule); e. narrow internal auditory canal; f. internal auditory canal enlarged; g. inner ear ossification; h. cochlear, vestibular, and semicircular canal hypoplasia.

In total, 144 patients with sensorineural hearing loss (from unrelated families) were reached through the Otolaryngology Department of Chinese PLA General Hospital and included in this study. High-resolution temporal bone CT was available for all 144 recruited patients. Isolated MD was defined as a complex malformation in which the normal cochlear two and a half turn spirals were replaced by a hypoplastic coil of one and a half turns because of an absence of the interscalar septum. EVA was considered to be enlarged if its diameter was > 1.5 mm at the midpoint between the common crus and the external aperture of the vestibular aqueduct. Based on the image information in these 144 patients, isolated MD was identified in 28 patients (19.4%), EVA with MD was identified in 50 patients (34.7%), EVA without MD was identified in 50 patients (34.7%), and other types of IEM were identified in 16 patients (11.1%). The other types of IEM included six cases of cystic cochlea, dilated vestibule, and enlarged internal auditory canal, two cases of common cavity (a cystic cavity representing the cochlea and vestibule without showing any differentiation into cochlea and vestibule), semicircular canal aplasia, and narrow internal auditory canal, one case of semicircular canal hypoplasia, one case of inner ear ossification, one case of vestibule and semicircular ossification, three cases of common cavity and enlarged internal auditory canal, one case of common cavity, and one case of common cavity with narrow internal auditory canal (Figure 1). Of the 144 patients, 82 were males and 62 were females. Ages ranged from 10 months to 25 years. In isolated MD group, ages ranged from 14 months to 26 years and the average age was 8 years. In EVA with MD group, ages ranged from 8 months to 29 years and the average age was 11 years. In EVA group, ages were from 9 months to 31 years and the average age was 10 years, and in IEM group, ages ranged from 15 months to 27 years and the average age was 10 years.

### Clinical evaluation

A complete history, physical and otoscopic examinations, and audiological testing, including pure tone audiometry, tympanometry, or auditory brainstem response, were carried out in all patients. No patient was found to have any goiter symptom or sign or syndromic symptoms in other systems. Pure-tone average (PTA) was calculated as the average of the threshold measured at 0.5, 1.0, 2.0, and 4.0 kHz and was used to compare subgroups of patients. The level of hearing loss, in terms of PTA, was described as follows: normal hearing, < 20 dB; mild hearing impairment, 21-40 dB; moderate hearing impairment, 41-70 dB; severe hearing impairment, 71-90 dB; and profound hearing impairment, > 91 dB.

### Mutational analysis

Informed consent was obtained from all participants or the parents of minors prior to blood sampling. This study was performed according to the protocol approved by the Ethics Committee of the Chinese PLA General Hospital.

DNA was extracted from peripheral blood leukocytes using a commercially available DNA extraction kit (Watson Biotechnology, Inc., Shanghai, China). DNA sequence analysis of the *SLC26A4 *gene was performed by polymerase chain reaction amplification of the 21 coding exons plus approximately 50-100 bp of the flanking intron regions with previously published primers [16]. The reaction mixture contained 100 ng DNA, 1.5 units of DNA Taq polymerase(TaKaRa, Dalian, China), 200 uM dNTPs, 3 pmol of each forward and reverse primer, 2.5 ul of 10 × buffer(with 2.5 mM MgCl_2_) and the final reaction volume was filled to 25ul with ddH_2_O. The exons in the gene were amplified according to the PCR conditions described previously [17,18]. The PCR amplified products were sequenced and analyzed using an ABI 3130 DNA sequencing machine (ABI, Foster City, CA, USA) and the ABI 3130 Analysis Software (v.3.7 NT) according to the manufacturer's instructions.

### Statistical testing

The distribution of *SLC26A4 *in the different groups belongs to nonparametric randomized block design information. The Friedman rank sum test (M-test) was used to determine whether there was a significance difference in the observed number of mutant *SLC26A4 *genes in the isolated MD, EVA with MD, EVA, and IEM groups [19]. The chi-squared test(R × C table) was used to compare the hearing level among the four groups[20]. All statistical methods were analyzed on SPSS (statistical package for the Social Sciences)15.0 software. A P-value of 0.05 or less was accepted as statistically significant.

## Results

The hearing loss phenotype ranged from moderate to profound. Table 1 shows the distribution of hearing impairment in the isolated MD, EVA with MD, EVA, and IEM groups. Most patients in the four groups had severe to profound hearing loss, but there was no difference in the distribution of hearing level among the four groups.

**Table 1:** Hearing levels in the different groups

Hearing level	MD	EVA with MD	EVA	IEM
Moderate	2	5	4	1
Severe	6	17	14	5
Profound	20	28	32	10

MD: Isolated Mondini dysplasia, EVA with MD: enlarged vestibular aqueduct with Mondini dysplasia, EVA: enlarged vestibular aqueduct without Mondini dysplasia, IEM: other types of inner ear malformations

DNA sequence analysis of *SLC26A4 *was performed in all 144 patients. Biallelic, monoallelic, and no *SLC26A4 *mutations were detected in 92 patients (63.9%), nine patients (6.3%), and 43 patients (29.9%), respectively. In the different groups, the detection rate of the *SLC26A4 *mutation was discrepant (Table 2).

**Table 2:** Distribution of *SLC26A4 *mutations in the different groups

Inner ear image	No mutation	Monoallelic mutation	Biallelic mutation	Total
MD	27 (96.4%)	1 (3.6%)	0	28 (100%)
EVA with MD	1 (2.0%)	3 (6.0%)	46 (92.0%)	50 (100%)
EVA	1 (2.0%)	3 (6.0%)	46 (92.0%)	50 (100%)
IEM	14 (87.5%)	2 (12.5%)	0	16 (100%)

Total	43	9	92	144

MD: Isolated Mondini dysplasia, EVA with MD: Enlarged vestibular aqueduct with Mondini dysplasia, EVA: enlarged vestibular aqueduct without Mondini dysplasia, IEM: Other types of inner ear malformations

In the isolated MD group, only one monoallelic mutation in *SLC26A4 *(c.919-2A>G, aberrant splicing) was found in one patient (1/28, 3.6%), and no mutation was found in the other 27 patients (27/28, 96.4%). In the EVA with MD group, biallelic, monoallelic, and no *SLC26A4 *mutations were identified in 46 patients (46/50, 92.0%), three patients (3/50, 6.0%), and one patient (1/50, 2.0%), respectively. In the EVA groups, biallelic, monoallelic, and no *SLC26A4 *mutations were identified in 46 patients (46/50, 92.0%), three patients (3/50, 6.0%), and one patient (1/50, 2.0%), respectively. Similar to the isolated MD group, only two patients were identified to carry monoallelic mutations of the *SLC26A4 *gene in the IEM group (2/16, 12.5%). Among them, one patient carried the c.919-2A>G mutation, and the other carried the c.1286C>A(p.A429E) mutation. All variants of *SLC26A4 *found in the EVA plus MD group and EVA group are listed in Table 3.

**Table 3:** Distribution of SLC26A4 genotypes in patients with different CT scan phenotype

CT phenotype	Patient number	Genotype of *SLC26A4*			
		
		allele 1		allele 2	
		
		nucleotide change	amino acid change	nucleotide change	amino acid change
**Isolated MD**	1	c.919-2 A>G[21]	aberrant splicing	-	-
**(1-28)**	2-28	-	-	-	-
					
**IEM**	29	c.919-2 A>G	aberrant splicing	-	-
**(29-44)**	30	1286C>A (this study)	A429E	-	-
	31-44	-	-	-	-
					
**EVA**	45	c.917insG(6)	Frameshift	-	-
**(45-94)**	46-57	c.919-2 A>G	aberrant splicing	c.919-2 A>G	aberrant splicing
	58	c.1226G>A[22]	R409H	c.1226G>A	R409H
	59	c.919-2 A>G	aberrant splicing	c.1520delT(this study)	Frameshift
	60	c.919-2 A>G	aberrant splicing	c.1343C>A[23]	S448*
	61	c.1174A>T[24]	N392Y	c.1975G>C[25]	V659L
	62-65	c.919-2 A>G	aberrant splicing	c.2168A>G[21]	H723R
	66	c.1229C>T[26]	T410M	c.1079C>T[27]	A360V
	67	c.2027T>A[23]	L676Q	c.ivs15+5G>A[26]	aberrant splicing
	68,69	c.919-2 A>G	aberrant splicing	c.1229C>T	T410M
	70	c.919-2 A>G	aberrant splicing	c.1829C>A[21]	S610*
	71,72	c.919-2 A>G	aberrant splicing	c.1343C>T[23]	S448L
	73	c.1229C>T	T410M	c.1594AG>T (this study)	SF532, 545*
	74,75	c.919-2 A>G	aberrant splicing	c.1975G>C	V659L
	76,77	c.919-2 A>G	aberrant splicing	c.946G>T(6)	G316X
	78	c.919-2 A>G	aberrant splicing	c.ivs9+1G>A (this study)	aberrant splicing
	79,80	c.919-2 A>G	aberrant splicing	c.1586T>G[23]	I529S
	81	c.2168A>G	H723R	-	-
	82	c.2168A>G	H723R	c.2168A>G	H723R
	83	-	-	-	-
	84	c.919-2 A>G	aberrant splicing	c.281C>T[23]	T94I
	85	c.919-2 A>G	aberrant splicing	-	-
	86	c.919-2 A>G	aberrant splicing	c.1318A>T[23]	K440*
	87	c.919-2 A>G	aberrant splicing	c.563T>C(this study)	I188T
	88	c.919-2 A>G	aberrant splicing	c.496delA (this study)	Frameshift
	89	c.919-2 A>G	aberrant splicing	c.1318A>T	K440*
	90	c.812A>G[23]	D271G	c.1225C>T (this study)	R409C
	91	c.919-2 A>G	aberrant splicing	c.1548insC[26]	Frameshift
	92	c.919-2 A>G	aberrant splicing	c.1173C>A (this study)	S391R
	93	c.919-2 A>G	aberrant splicing	c.1174A>T	N392Y
	94	c.2168A>G	H723R	c.2T>C[16]	M1T
**EVA with MD**	95	-	-	-	-
**(95-140)**	96-99	c.919-2 A>G	aberrant splicing	c.2168A>G	H723R
	100-102	c.919-2 A>G	aberrant splicing	c.589G>A[23]	G197R
	103	c.2168A>G	H723R	c.1594AG>T	SF532, 545*
	104-115	c.919-2 A>G	aberrant splicing	c.919-2 A>G	aberrant splicing
	116	c.919-2 A>G	aberrant splicing	c.1238A>G[28]	Q413R
	117	c.919-2 A>G	aberrant splicing	c.414delT (this study)	Frameshift
	118	c.1229C>T	T410M	c.1343C>A	S448*
	119	c.2168A>G	H723R	c.754T>C[26]	S252P
	120,121	c.919-2 A>G	aberrant splicing	c.ivs13+9C>T[29]	aberrant splicing
	122,123	c.919-2 A>G	aberrant splicing	c.1229C>T	T410M
	124	c.1174A>T	N392Y	c.2027T>A	L676Q
	125-127	c.919-2 A>G	aberrant splicing	c.1975G>C	V659L
	128	c.2168A>G	H723R	c.1079C>T	A360V
	129,130	c.919-2 A>G	aberrant splicing	c.2027T>A	L676Q
	131	c.919-2 A>G	aberrant splicing	c.1340delA(this study)	Frameshift
	132	c.1174A>T	N392Y	-	-
	133	c.919-2 A>G	aberrant splicing	c.218delA(this study)	Frameshift
	134	c.2168A>G	H723R	c.2086C>T(this study)	Q696*
	135	c.ivs15+5G>A	aberrant splicing	c.1520delT	Frameshift
	136,137	c.919-2 A>G	aberrant splicing	c.1226G>A	R409H
	138,139	c.919-2 A>G	aberrant splicing	-	-
	140	c.919-2 A>G	aberrant splicing	c.1991C>T(this study)	A664V
	140	c.2168A>G	H723R	c.439A>G[21]	M147V
	140	c.919-2 A>G	aberrant splicing	c.1594A>C[23]	S532R
	140	c.919-2 A>G	aberrant splicing	c.ivs15+5G>A	aberrant splicing
	140	c.1174A>T	N392Y	c.1174A>T	N392Y

Isolated MD group: Patient 1 had only one mutation and patient 2-28 had no mutation in *SLC26A4*. IEM group: Patient 29, 30 had one mutation and patient 31-44 had no mutation in *SLC26A4*. EVA group: Most patients had bi-allelic mutations except Patient 45, 81, 85 had one mutation and patient 83 had no mutation in *SLC26A4*. EVA with MD group: Most patients had bi-allelic mutations except Patient 132, 138, 139 had one mutation and patient 95 had no mutation.

**Figure 2:**
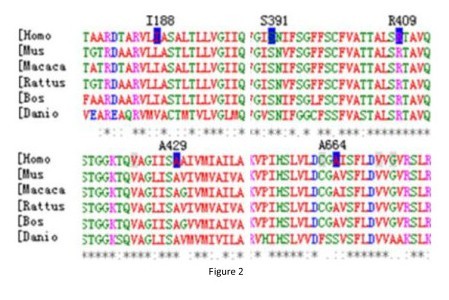
**Sequence alignment of partial amino acid sequence of human *SLC26A4 *and its homologs**. Positions of mutations are indicated in blue.

In this study, in total, 39 *SLC26A4 *mutations were found among the 144 patients. Most *SLC26A4 *mutations (97%, 38/39) were found in the EVA group and the EVA with MD group, with the exception of one single allelic mutation (c.919-2A>G) in the isolated MD group and two single allelic mutations (c.919-2A>G and c.1286C>A) in the group with other IEM. Some mutations observed in present work, as indicated in Table 3 were previously reported to be pathogenic. Among these previously reported mutations, c.919-2A>G (51%, 102/200) was the most prevalent in the EVA with MD and EVA groups. The mutations p.H723R, p.T410M, p.V659L, and p.N392Y were also more common than the others in both groups (Table 3). Thirteen variants had not been previously reported (Table 3), consisting of seven missense variants (c.1286C>A(p.A429E), c.1594AG>T(SF532,545*), c.563T>C (p.I188T), c.1225C>T(p.R409C), c.1173C>A(p.S391R), c.2086C>T(p.Q696*), and c.1991C>T (p.A664V)), five deletions (c.1520delT, c.496delA, c.414delT, c.1339-1340delA, and c.218delA), and one splice site variant (c.ivs9+1G>A). Among the novel variants, c.1594AG>T(SF532,545*), c.2086C>T(p.Q696*), c.1520delT(SF507,507*), c.496delA(SF166, 171*), c.414delT(SF138, 144*), c. 1340delA(SF447, 454*), and c.218delA(SF73, 96*) encode either frameshift or nonsense mutations, resulting in early translational termination, severe truncation of the pendrin polypeptide, and, very likely, non-functional and/or unstable polypeptide products. The novel splice site (c.ivs9+1G>A) and five missense mutations (p.A429E, p.I188T, p.R409C, p.S391R, p.A664V) affect residues that are conserved among *SLC26A4 *orthologs [Mus (mouse), Macaca, Rattus, Bos, Danio; Figure 2]. These mutations were not present in a screen of 50 patients without IEM and 200 normal Chinese controls in Yuan's research [6], suggesting that these five mutations are also likely to be pathogenic.

The statistical analysis showed that there were significant differences in the frequency of *SLC26A4 *mutation among the groups (P < 0.001). In the isolated MD group, only one single allelic mutation in *SLC26A4 *was found in one patient (1/28, 3.6%). In the EVA with MD group, 46 patients (46/50, 92.0%) carried biallelic mutations, and three patients carried single allelic mutations (3/50, 6.0%). In the EVA group, 46 patients (46/50, 92.0%) had biallelic mutations and three patients with one mutation (3/50, 6.0%). Only two patients carried single allelic mutations of the *SLC26A4 *gene in the IEM group (2/16, 12.5%). The distribution of *SLC26A4 *mutation in the isolated MD group was significantly lower than that in the EVA with or without MD patients (P < 0.001), suggesting that mutations in the *SLC26A4 *gene are more frequently associated with the EVA phenotype. There was no significant difference between the isolated MD and IEM groups (P > 0.5) and no difference in mutation distribution between the EVA with MD group and EVA group (P > 0.5). Yuan's research [6] identified four carriers of heterozygous c.919-2A>G among 200 Chinese individuals with normal hearing. There was no significant difference in mutation distribution between isolated MD patients and Yuan's research (P > 0.5).

## Discussion

DNA sequence analysis for *SLC26A4 *mutations was performed in 144 patients with sensorineural hearing loss in this study. Inner ear malformation was present presented in 144 patients, including isolated MD, EVA with MD, isolated EVA (without MD), and other IEM identified on temporal bone CT scan images. The mutation detection rates of *SLC26A4 *were 3.6%, 98%, 98%, and 12.5% in the isolated MD group, EVA with MD group, EVA group, and IEM group, respectively. The prevalences of biallelic mutations in *SLC26A4 *were 0%, 92%, 92%, and 0%, and prevalences of monoallelic mutations were 3.6%, 6.0%, 6.0%, and 12.5% in the four respective aforementioned groups. The detection rate of *SLC26A4 *mutations in patients with EVA with or without MD in this study were consistent with the frequencies in other Asian studies, which are 87%, 97.9%, 92%, and 78% in Taiwanese, Chinese, Japanese, and Korean EVA patients, respectively [6,21,26,30]. Meanwhile, the mutation detection rate of this gene in Caucasian EVA patients is much lower, at 53% and 40%, respectively, in the UK and Europe [31,32]. In the US population, mutations in *SLC26A4 *account for about one-third of EVA patients [33]. In these studies, however, the researchers did not clearly define the specific concept of Mondini.

Azaiez [34] presented a comprehensive analysis of 474 patients from the Molecular Otolaryngology Research Laboratories of the University of Iowa Hospital and Clinics (most patients were Caucasians) with temporal bone anomalies to determine the correlation between *SLC26A4 *genotypic and phenotypic data. In Azaiez's study, Mondini was diagnosed a complex malformation that includes a cochlear dysplasia and EVA (EVA with MD). This study showed that 24.1% (28/116) of patients diagnosed EVA with MD had biallelic mutations in *SLC26A4*, but only 11.1% (38/341) of patients with isolated EVA had biallelic mutations in *SLC26A4*. There was a statistically significant difference in mutation distribution between isolated EVA patients and EVA with MD patients, with the presence of mutations in *SLC26A4 *more frequently associated with the EVA with MD phenotype. Azaiez suggested that mutations in *SLC26A4 *were more likely to be of a genetic etiology in EVA with MD than in EVA, in the other side of the spectrum, the environment is believed to play a bigger role in isolated EVA patients. In Campbell's research [35], 57% of patients from the US with Mondini (EVA with MD) from simplex families carried *SLC26A4 *mutations, and he considered that *SLC26A4 *mutations were present mostly in patients with EVA plus MD. The observations in the present study were not consistent with either Azaiez or Campbell's findings. There was no difference in the distribution or mutation frequency between the EVA group and EVA with MD group in our study. The mutation detection rate in *SLC26A4 *was 98% in both the EVA with MD group and the EVA group, and the percentage of patients with biallelic mutations was 92% and in both groups.

*SLC26A4 *encodes pendrin, an anion transporter protein [10]. In the inner ear, pendrin is expressed in cells of the external sulcus, epithelial cells of the endolymphatic duct and sac, and nonsensory cells at the margin of the maculae of the utricle and saccule. In all of these cell types, the apical surface is exposed to endolymph, consistent with pendrin's role as an anion transporter. The variant of pendrin was able to exchange anions between the cytosol and extracellular fluid [36]. Table 3 shows that the *SLC26A4 *mutations c.919-2A>G and p.H723R were highly prevalent in both in the EVA with MD and EVA groups, and the mutation spectrums were also very similar between the two groups. Our data provides strong evidence that the molecular pathogenesis of EVA with or without MD were identical solely because of the similar ORF mutation spectrum in *SLC26A4 *in the Chinese population and that the ORF mutations in *SLC26A4 *were not likely to be the reason for the pathogenesis of isolated MD in this Chinese population. So it is reasonable that there were the same mutation detection rates of SLC26A4 in both the EVA with MD group and the EVA group in this study.

The most significant mechanistic insights into the pathogenesis of hearing loss associated with EVA are based upon the *SLC26A4 *knockout (*SLC26A4*-/-) mouse that segregates a targeted deletion of exon 8 of *SLC26A4 *[37] and the loop mouse line segregating a chemically induced mutation of *SLC26A4 *[38]. In the mouse inner ear, pendrin is expressed in the cochlea, the vestibular labyrinth and the endolymphatic sac and pendrin functions as a Cl^-^/HCO_3_^- ^exchanger. The initial pathologic alterarion in *SLC26A4*-/- mice includes an enlargement of the endolymphatic sac and cochlea that develops at E14.5 [39]. Studies of an *SLC26A4 *knockout mouse demonstrate that acidification and enlargement of the scala media are early events in the pathogenesis of deafness. The enlargement is driven by fluid secretion in the vestibular labyrinth and a failure of fluid absorption in the embryonic endolymphatic sac.

Our data showed that the distribution of *SLC26A4 *mutation in the isolated MD group was significantly lower than that in the EVA with or without MD patients; the one patient from the MD group who carried a monoallelic mutation of *SLC26A4 *(c.919-2A>G) might have been a coincident carrier of this mutation. There was no significant difference among the MD group, IEM group (P > 0.5), and normal controls (previous data published by Yuan [6]). Our results did not show a relationship between isolated MD and the *SLC26A4 *gene in this Chinese population. The hearing impairment in our MD group might have been caused by factors other than the *SLC26A4 *gene in our Chinese population.

The development of the human ear begins around the fourth week of embryonic life. The inner ear consists of two components: a membranous and a bony labyrinth. The membranous labyrinth is derived from the ectoderm, whereas the bony labyrinth/otic capsule is derived from the mesoderm and neural crest surrounding the primordial membranous labyrinth. The primordium of the cochlear duct evaginates from the ventral portion of the otocyst starting at the fifth week. This evagination extends anteromedially and gradually begins to coil. By the eighth week, the cochlea has formed 1.5 turns, and at the tenth week, 2 turns; at the 25^th ^week, the cochlear duct has acquired the mature 2.5 turns. Ossification of the cartilaginous capsule to form the bony labyrinth does not occur until the membranous labyrinth has acquired its adult size. Bone formation starts at the 15^th ^week and ends by the 21^st ^week, with a total of 14 ossification centers [40]. In cochlear Mondini, it is thought that the arrest of development is at the seventh week of gestation. During inner ear development, many genes causing IEM and deafness have been identified, including *SLC26A4*, *EYA1 *[41], *FGFR3 *[42,43], and *TCOF1 *[44,45]. Pathological analysis performed on hearing impaired patients revealed the presence of Mondini dysplasia, characerized by a reduced number of cochlear turns and a incomplete interscalar sptum [46,47]. Mouse models have made it possible to study the molecular origins of the ear pathology found in both VCFS/DGS and DFN3. Mice homozygous null for Tbx1 die at birth and exhibit severe ear defects due to early arrest of inner ear development [48]. Additionally, some Brn4 null mice display a reduction in cochlear coiling. The normal mouse cochlea consist of 1.75 turns; however, approximately 25% of Brn4 null mice have less than 1.5 turns, indicating a role for Brn4 in epithelial-mesenchymal interactions during ear development[49]. With the exception of the aforementioned genes, it was evident to clinicians that retinoids (vitamin A) or other factors had potent teratogenic effects in isolated MD patients when administered early in pregnancy [50]. These factors increase the complexity of determining the real etiology of isolated MD, and our observations hint that ethnic background is apparently important and may affect the etiologic explanation of isolated MD. We suggest that EVA with or without MD and isolated MD in this Chinese population have totally different pathogenic mechanisms.

## Conclusion

We did not review the relationship between *SLC26A4 *and isolated MD in a Chinese deaf population, but we provided evidence to support the idea that mutations in *SLC26A4 *were the most important factor in the pathogenesis of EVA with and without MD. This will facilitate genetic testing and counseling in patients with different types of inner ear malformation, and may provide motivation to explore the real etiology of isolated MD by new genomic strategies and platforms, such as capturing and sequencing all possible genes related to the development of the inner ear using next-generation sequencing technology.

## Competing interests

The authors declare that they have no competing interests.

## Authors' contributions

SH carried out the molecular genetic studies, participated in the sequence alignment and drafted the manuscript, and participated in the design of the study and performed the statistical analysis. YY and GW carried out temporal CT scan and thyroid hormone assays. D K, XZ, XM and XY participated in the sequence alignment. MD carried out epidemiological survey. DH and PD conceived of the study, and participated in its design and coordination and helped to draft the manuscript. All authors read and approved the final manuscript.
